# C-reactive protein, immunothrombosis and venous thromboembolism

**DOI:** 10.3389/fimmu.2022.1002652

**Published:** 2022-09-13

**Authors:** Caroline Dix, Johannes Zeller, Hannah Stevens, Steffen U. Eisenhardt, Karen S. Cheung Tung Shing, Tracy L. Nero, Craig J. Morton, Michael W. Parker, Karlheinz Peter, James D. McFadyen

**Affiliations:** ^1^Department of Haematology, Alfred Hospital, Melbourne, VIC, Australia; ^2^Australian Centre for Blood Diseases, Monash University, Melbourne, VIC, Australia; ^3^Atherothrombosis and Vascular Biology Program, Baker Heart and Diabetes Institute, Melbourne, VIC, Australia; ^4^Department of Plastic and Hand Surgery, University of Freiburg Medical Centre, Medical Faculty of the University of Freiburg, Freiburg, Germany; ^5^Department of Biochemistry and Pharmacology, Bio21 Molecular Science and Biotechnology Institute, The University of Melbourne, Parkville, VIC, Australia; ^6^Baker Department of Cardiometabolic Health, The University of Melbourne, Parkville, VIC, Australia; ^7^Commonwealth Scientific and Industrial Research Organisation (CSIRO) Biomedical Manufacturing Program, Clayton, VIC, Australia; ^8^Structural Biology Unit, St. Vincent’s Institute of Medical Research, Fitzroy, VIC, Australia; ^9^Department of Cardiology, Alfred Hospital, Melbourne, VIC, Australia

**Keywords:** C-reactive protein, venous thromboembolism, immunothrombosis, COVID-19, Thromboinflammation

## Abstract

C-reactive protein (CRP) is a member of the highly conserved pentraxin superfamily of proteins and is often used in clinical practice as a marker of infection and inflammation. There is now increasing evidence that CRP is not only a marker of inflammation, but also that destabilized isoforms of CRP possess pro-inflammatory and pro-thrombotic properties. CRP circulates as a functionally inert pentameric form (pCRP), which relaxes its conformation to pCRP* after binding to phosphocholine-enriched membranes and then dissociates to monomeric CRP (mCRP). with the latter two being destabilized isoforms possessing highly pro-inflammatory features. pCRP* and mCRP have significant biological effects in regulating many of the aspects central to pathogenesis of atherothrombosis and venous thromboembolism (VTE), by directly activating platelets and triggering the classical complement pathway. Importantly, it is now well appreciated that VTE is a consequence of thromboinflammation. Accordingly, acute VTE is known to be associated with classical inflammatory responses and elevations of CRP, and indeed VTE risk is elevated in conditions associated with inflammation, such as inflammatory bowel disease, COVID-19 and sepsis. Although the clinical data regarding the utility of CRP as a biomarker in predicting VTE remains modest, and in some cases conflicting, the clinical utility of CRP appears to be improved in subsets of the population such as in predicting VTE recurrence, in cancer-associated thrombosis and in those with COVID-19. Therefore, given the known biological function of CRP in amplifying inflammation and tissue damage, this raises the prospect that CRP may play a role in promoting VTE formation in the context of concurrent inflammation. However, further investigation is required to unravel whether CRP plays a direct role in the pathogenesis of VTE, the utility of which will be in developing novel prophylactic or therapeutic strategies to target thromboinflammation.

## Introduction

C-reactive protein (CRP) is an acute phase reactant widely used in clinical practice as a marker of infection and/or inflammation owing to the fact that its synthesis rapidly and dramatically (up to 10,000-fold) increases after tissue injury or infection (1). However, a significant body of work exists demonstrating that destabilized isoforms of CRP possess important pro-inflammatory and pro-thrombotic properties and can therefore amplify pathological conditions associated with ‘immunothrombosis’ (2, 3). It is now appreciated that venous thromboembolism (VTE) represents a prototypical immunothrombotic response. Accordingly, VTE risk is significantly elevated in a number of conditions associated with inflammation and elevations of plasma CRP levels, particularly surgery, obesity, sepsis, cancer, inflammatory bowel disease (IBD) and COVID-19. As will be discussed below, despite evidence highlighting a direct role of CRP in amplifying thromboinflammatory responses in the setting of ischemia/reperfusion injury and transplant rejection, the direct causal role of CRP in VTE pathogenesis has not yet been thoroughly investigated. However, a significant body of research has focused on whether CRP is associated with VTE in a variety of clinical contexts. This review will discuss potential mechanisms by which CRP may play a direct role in mediating VTE pathogenesis, the clinical evidence for the role of CRP in predicting first episode VTE, recurrent VTE and in specific subsets of patients such as those with COVID-19 and cancer-associated thrombosis (CAT).

## CRP structure and function

CRP is a member of the highly evolutionary conserved pentraxin superfamily of proteins and is composed of five identical protomers arranged in a pentameric configuration, termed pentameric CRP (pCRP) (4). Importantly, each CRP protomer contains a phosphocholine (PC) binding site which allows the binding of pCRP to PC exposed on inflamed, apoptotic cells and bacterial cell walls. The PC binding pockets of pCRP, contained on the ‘binding’ face of the CRP pentamer, include two critical residues for mediating CRP-PC interactions. These residues, Phe-66 and Glu-81, mediate the interactions between the methyl group and the positively charged nitrogen of PC, respectively (5). The opposite face of the CRP pentamer is the ‘effector’ face, and it has the ability to bind to, and interact with, the globular head of the complement C1q subunit and Fcγ receptors, thus endowing CRP with the ability to regulate innate immune functions (6). Intriguingly, pCRP is now understood to be a circulating precursor form of the ‘CRP system’, and it is not a pro-inflammatory molecule. In support, it has been demonstrated that the infusion of purified pCRP in healthy human volunteers does not induce pro-inflammatory effects (7).

**Figure 1:**
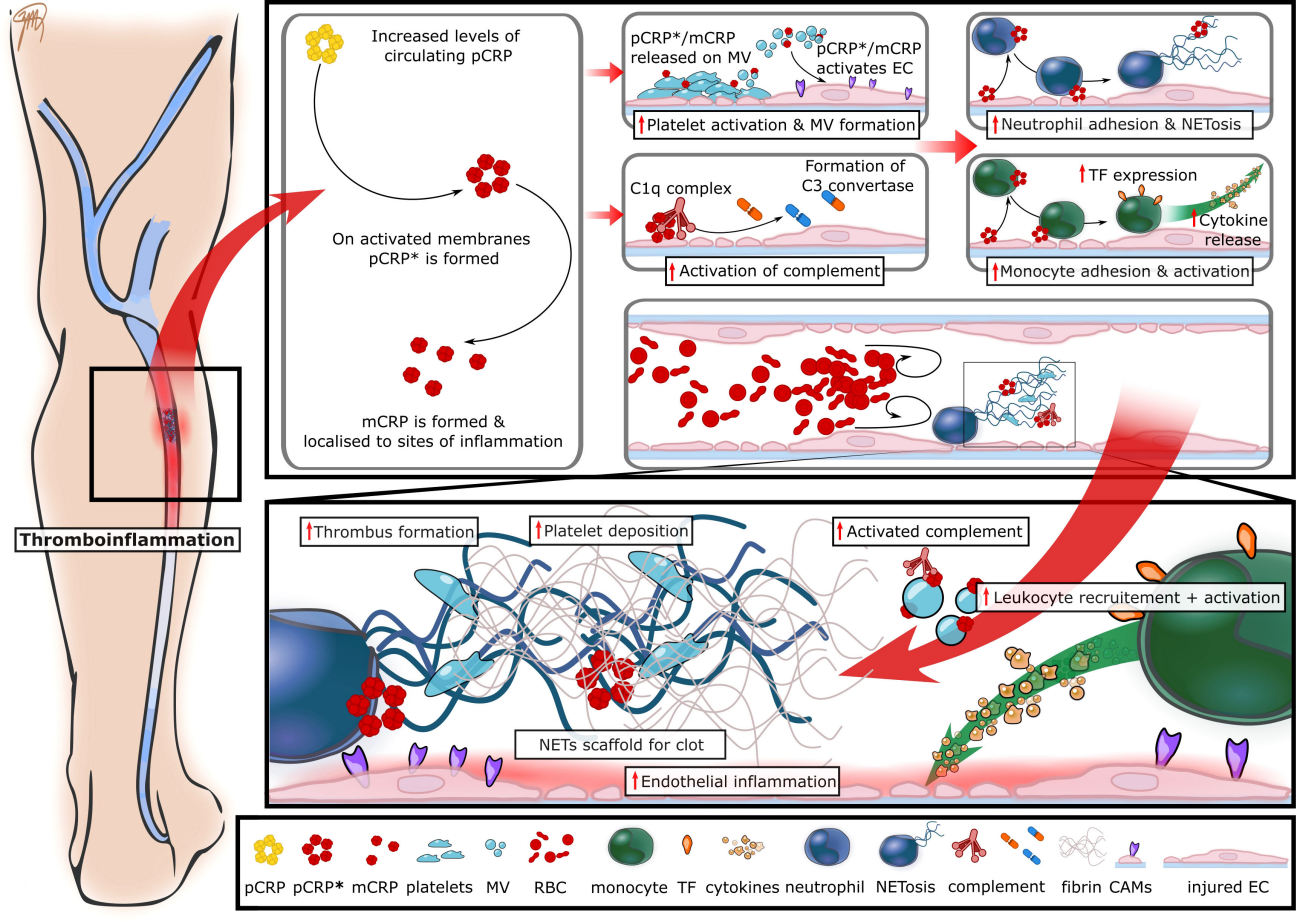
The proposed role of CRP in immunothrombosis and VTE pathogenesis. The inciting events leading to VTE formation are endothelial inflammation with the subsequent recruitment of platelets and leukocytes. Leukocytes provide a source of tissue factor (TF) and highly activated neutrophils undergo NETosis, thereby resulting in thrombin formation and the generation of fibrin. Circulating CRP levels rapidly increase in the setting of inflammation and pCRP binds to disturbed cell surfaces, such as inflamed endothelial cells (EC) and activated platelets where it dissociates into the pro-inflammatory and pro-thrombotic isoforms pCRP* and mCRP. These pro-inflammatory isoforms amplify further activation of the endothelium, platelets and bind to complement C1q, thereby initiating complement activation. These changes result in further recruitment and activation of leukocytes ultimately leading to increased tissue TF and NET formation, thus resulting in enhanced thrombus formation. CAMs, cell adhesion molecules; EC, endothelial cells; MV, microvesicles; RBC, red blood cells; CRP, C-reactive protein; pCRP, pentameric CRP; pCRP*, neoepitope expressing pCRP; mCRP, monomeric CRP; NETs, neutrophil extracellular traps; TF, tissue factor.

The biological function of CRP appears significantly dependent upon conformational changes that can occur in response to a range of stimuli including binding to PC-rich membranes, exposure to an acidic microenvironment, heat or urea, or binding to misfolded proteins such as beta amyloid (3, 8). Indeed, our group recently demonstrated that upon binding of pCRP to PC-enriched membranes with curvature similar to that observed for platelet, endothelial and leukocyte derived microvesicles, pCRP relaxes its overall pentameric conformation to form pCRP* (2, 9). This structural destabilization initiates the exposure of neoepitopes at the interface of adjacent CRP protomers, thus allowing the binding of C1q to the central pore of pCRP* with subsequent activation of the classical complement pathway (**Figure 1**) (3, 4).

Although yet to be precisely delineated, it is currently thought that pCRP* is an ‘intermediate’ conformation that precedes dissociation of the CRP protomers and the formation of monomeric CRP (mCRP) (**Figure 1**). The formation of mCRP exposes a range of interprotomer neoepitopes which are thought to account for its potent pro-inflammatory and pro-thrombotic functions (2, 10). Interestingly, the dissociation of pCRP to mCRP is associated with a shift in solubility such that mCRP is relatively insoluble and therefore it is likely that this isoform does not circulate as an unbound protein in blood but rather remains anchored on activated cellular membranes, e.g. on activated platelets or extracellular vesicles (2, 3, 9, 11). These observations have resulted in the concept that pCRP and pCRP* may represent the circulating forms of CRP, whilst mCRP is likely to be generated at sites of inflammation and is predominantly tissue bound.

## The concept of immunothrombosis and the pro-thrombotic and pro-inflammatory properties of pCRP* and mCRP

It is now well appreciated that venous thrombosis is a consequence of thromboinflammation. Indeed, seminal work by von Bruhl et al. demonstrate that the initial stages of VTE pathogenesis are incited by endothelial inflammation which facilitates the subsequent recruitment of platelet and leukocytes – predominantly neutrophils and monocytes (**Figure 1**) (12). These events culminate in the activation of recruited cells and ultimately conspire in the formation of neutrophil extracellular traps (NETs) through the process of NETosis (13–15). The formation of NETs triggers fibrin formation *via* activation of the contact pathway of coagulation, whilst monocytes bearing tissue factor (TF) initiate the extrinsic coagulation pathway (12). These findings demonstrate the inextricable link between the immune and coagulation systems, and led to the concept of “immunothrombosis”, an innate immune response induced by intravascular thrombus formation leading to the recognition and destruction of pathogens (16). Significantly, the pro-inflammatory and pro-thrombotic isoforms of CRP, i.e. pCRP* and mCRP, have demonstrated significant biological effects in regulating many of the aspects central to pathogenesis of atherothrombosis and VTE (17).

Indeed, pCRP* and mCRP have been demonstrated to induce endothelial inflammation and facilitate leukocyte adhesion and transmigration *in vivo* (18, 19). *In vitro*, the application of mCRP to endothelial cells inhibits nitric oxide production and increases the production of the pro-inflammatory chemokines interleukin-8 (IL-8) and monocyte chemoattractant protein-1 (MCP-1) in an NF-κB dependent manner (20). Moreover, the expression of the key adhesion molecules, vascular cell adhesion protein-1 (VCAM-1) and intercellular adhesion molecule-1 (ICAM-1), are upregulated in response to mCRP stimulation, both of which serve to facilitate leukocyte adhesion (18, 19). Importantly, the pro-inflammatory isoforms of CRP not only promote leukocyte adhesion but also serve to enhance leukocyte activation. mCRP can directly stimulate monocytes and neutrophils resulting in activation of the leukocyte integrin αMβ2 (Mac-1), which is a critical binding partner for glycoprotein Ib (GP-Ib) on platelets (21, 22). As such, mCRP acts to enhance leukocyte recruitment and adhesion to the vasculature, consolidates leukocyte-platelet aggregation, and amplifies the local inflammatory responses. Critically in the context of VTE, CRP has been demonstrated to upregulate monocyte TF expression in addition to inducing NET formation (12, 23). These steps are critical in VTE pathogenesis since, genetic deletion, or pharmacological inhibition of these processes afford significant protection from VTE formation in animal models (12).

In addition to its effects on endothelial cells and leukocytes, mCRP can contribute to thrombosis by way of its ability to activate platelets. Although the receptor responsible for mediating mCRP-induced platelet activation remains to be delineated, a role for the platelet scavenger receptor, CD36, has been suggested (24). mCRP stimulation of platelets leads to activation of the major platelet adhesion receptor, GPIIb/IIIa, and results in alpha granule exocytosis (24). These functional responses promote thrombogenesis by enhancing platelet aggregation and leukocyte recruitment. Interestingly, platelet thrombi can also act as a scaffold for CRP dissociation which in turn acts to enhance thrombus growth (24, 25). Moreover, microvesicles enriched in lysophosphatidylcholine, and derived from activated platelets and monocytes, can bind to and destabilize pCRP to produce the pro-inflammatory CRP isoforms and induce inflammatory responses in endothelial cells (2, 9). Furthermore, activated platelets can interact directly with neutrophils and cause NET formation (26).

As described above, neoepitope-expressing pCRP* can bind the C1q subunit and trigger activation of the classical complement pathway. Well recognized as a fundamental component of the innate immune response, there is increasing evidence highlighting an important role for complement in thrombosis (27, 28). Indeed, complement and the coagulation pathways have likely evolved from a common ancestral pathway and share many similarities since both involve the activation of inactive zymogens into potent serine proteases. Recent data has highlighted how C1q can bind to, and activate platelets, in addition to interacting with von Willebrand Factor (vWF), to enhance platelet adhesion (29). As well as the direct effects of C1q on platelet responses, downstream products of C1q activation, namely the anaphylatoxins C3a and C5a, have been shown to promote thrombosis by activating endothelial cells and neutrophils and upregulating TF expression (27). Accordingly, population-based studies have linked elevated levels of C3 and complement activation pathways with an increased risk of VTE (30).

## Understanding the role of CRP in venous thromboembolism

VTE includes deep vein thrombosis (DVT) and pulmonary embolism and is associated with substantial mortality which, in contrast to arterial thromboembolic disease, has not improved in recent years (31). The historical understanding of venous thrombus formation is centred upon the mechanisms now well described as Virchow’s triad, which include vessel wall damage, venous stasis and hypercoagulability. However, mounting evidence demonstrates the critical importance of immunothrombosis and suggests that venous and arterial thrombotic disorders may be part of a continuous spectrum of disease. Indeed, it is now well appreciated that inflammation plays an important role in the pathogenesis of cardiovascular disease (32). In this regard, baseline circulating CRP levels in asymptomatic individuals constitute an independent risk factor for coronary (33) and peripheral vascular disease (34). The Women’s Health Study (35) and Physicians’ Health Study (36) both demonstrated that the circulating level of CRP is a predictor of future cardiovascular events, independent of other risk factors including hypertension, obesity and hypercholesterolemia. Additionally, short-term mortality post-acute myocardial infarct is associated with CRP responses (37). Possible explanations for these phenomena include the fact that circulating CRP levels reflect inflammation related to the atherosclerotic process and/or the extent of myocardial necrosis, and that CRP potentially directly interacts with atherosclerotic vessels (38). Indeed, preclinical studies have consistently demonstrated a convincing role for CRP, potentially *via* its activating isoforms, in directly amplifying tissue damage in rodent models of myocardial infarction (39). This highlights new therapeutic implications for cardiovascular and thromboinflammatory diseases (40).

Following the findings that circulating CRP levels predict cardiovascular events, and that statins lower the level of CRP in addition to cholesterol, the JUPITER study was devised to assess if rosuvastatin (compared to placebo) could reduce the risk of cardiovascular events in older individuals with normal cholesterol but high CRP (> 2 mg/L) (41, 42). The trial was stopped early due to a significant reduction in major cardiovascular events in the rosuvastatin arm (0.77 vs 1.36 per 100 person-years, hazard ratio (HR) 0.56) (41). Importantly, rosuvastatin also significantly reduced the occurrence of symptomatic VTE (0.18 vs 0.32 per 100 person-years of follow up, HR of 0.57) compared to placebo (43), as well as significantly reducing circulating CRP levels at 12 months follow up (2.2 vs 3.5 mg/L) (41, 43). As a result of these findings, there has been increasing interest in the value of circulating CRP levels in predicting VTE.

In addition to considering plasma levels of CRP, several studies have evaluated the interaction between genetic polymorphisms in the CRP gene and thrombotic risk. To date, studies pertaining to single nucleotide polymorphisms in the CRP gene and VTE do not suggest a significant association between genetically elevated CRP and VTE risk, which is in contrast to data regarding cardiovascular disease and CRP polymorphisms (44–46). Importantly, the evaluated polymorphisms in the CRP gene are not uniform across studies for arterial and venous thrombotic disease, and the utility of these genetic tests in predicting thrombotic risk is yet to be fully elucidated.

## CRP in acute VTE

Acute DVT is typically associated with a classic inflammatory response (redness, heat, swelling, pain, loss of function) which can manifest clinically at the location of the DVT in most patients (47). As such, circulating CRP levels have been measured to understand the utility of CRP as a biomarker in the clinical diagnosis of VTE, and to gain insight into the potential causal relationship between inflammation and thrombosis. In this acute setting, individuals with an acute DVT have higher circulating CRP levels compared to individuals who do not have DVT (48, 49), and a higher median CRP is associated with a significantly higher odds of DVT diagnosis (48). These findings appear to be particularly prominent in patients with proximal or extensive VTE, who are more likely to demonstrate a higher circulating CRP level compared with distal or smaller thromboses (47). In addition to these acute elevations at the time of DVT diagnosis, circulating CRP levels have been shown to be elevated in the 90-days prior to a diagnosed VTE episode, which highlights inflammation as a trigger for VTE (50). However, studies have also demonstrated that circulating CRP levels typically fall in the days following acute thrombosis diagnosis and anticoagulation initiation, which could indicate that inflammation and the rise in circulating CRP levels are the result of the thrombotic event rather than the cause (49). Further research is required to fully elucidate this complex interaction. Whilst these data do not demonstrate causality, they serve to further highlight the intimate link between inflammation and thrombosis in the context of VTE.

## CRP as a predictor of incident VTE

Although it is evident that acute VTE is associated with an increase in circulating CRP levels, whether long-term, low-grade CRP elevations predicts future VTE events, as it does with cardiovascular disease, remains controversial. Indeed, the results from a number of large, population-based studies examining this question have been conflicting, and associations found have typically been modest. These studies are summarized in **Table 1**. The differences may be partly explained by intra-individual variation in inflammatory markers, which is able to mask a true but small impact of low-grade inflammation on VTE risk, as well as significant variability in patient population by ethnicity, gender, and age (60). Additionally, timing of circulating CRP measurement in relation to the VTE episode was likely to play a role in the varied results, as well as the variation in the type of CRP assays used and their sensitivity. Moreover, given the growing awareness of the different biological activities of mCRP and pCRP*, it is important to highlight that no study to date has measured how different isoforms of CRP change in acute VTE.

**Table 1:** Summary of population-based studies assessing the relationship between circulating CRP levels and VTE.

Author, year of publication	Type of study & participant numbers	Patient group	Timing of CRP measurement	Risk association to first or recurrent VTE	Association (yes/no)
Ridker et al., 1997 (34)	Nested case control study; 101 VTE and 543 controls.	Male physicians	Baseline CRP, prospectively followed to subsequent VTE development.	Non-significantly higher CRP (1.26 vs 1.13 mg/L, p=0.34) in cases compared to controls.	No
Kamphuisen et al., 1999 (51)LETS study	Case control study; 474 VTE and 474 controls.	Age <70 years Excluded malignancy	CRP measured at least 6 months after VTE episode.	CRP higher in VTE cases (1.49 mg/L, 95% CI 1.32-1.68), compared to controls (1.12 mg/L, 95% CI 1.0-1.25).	Yes
Tsai et al., 2002 (52)LITE study (combined CHS and ARIC studies)	Cohort / nested case control study;21680 participants.	Middle aged and elderly.Excluded prior VTE or cancer.	Baseline CRP, prospective follow up (median 7.8 years).	No association between baseline CRP and subsequent VTE.	No
Vormittag et al., 2005 (53)	Case control study;214 VTE and 104 controls.	Unprovoked VTE Excluded malignancy, diabetes, IBD, HRT or rheumatic disease.	CRP measured after VTE episode (at least 3 months after diagnosis).	Non-significant adjusted OR for VTE of 1.7 (95% CI 0.7-4.5) per 1 mg/L increment in CRP. Unadjusted OR 2.8 (1.1-6.8).	No
Folsom et al., 2009 (54).ARIC study	Cohort study; 10505 participants.	Caucasian and African American aged 45-64 years.Excluded prior VTE or CRP >20 mg/L.	CRP at baseline, 8.3 years of prospective follow up.	HR for VTE 2.07 for those with CRP above 10th percentile (>8.55 mg/L) compared to lowest 90%.	Yes
Luxembourg et al., 2009 (55)MAISTHRO registry	Case control study;101 unprovoked VTE, 101 provoked VTE and 202 healthy controls.	Age 18-69 years Excluded those with VTE <3 months or >5.5 years prior and >1 prior VTE; malignancy, pregnancy, autoimmune disease in prior 3 months.	CRP taken after VTE episode.	Higher CRP in those with unprovoked VTE compared to provoked VTE and healthy controls.	Yes
Zacho et al., 2010 (44)CCHS and CGPS	2 cohorts:1. Prospective study, 10135 participants;2. Cross-sectional study, 36616 participants.	Danishgeneral population, no exclusions.	CCHS: Baseline CRP; 16 year prospective follow up.CGPS: VTE may have occurred before or after CRP measurement.	2.3x increased risk VTE if CRP >3 mg/L compared to <1 mg/L.No association between genetically increased CRP and VTE risk.	Yes
Quist-Paulsen et al., 2010 (56)HUNT-2 study	Nested case control study;515 cases and 1505 controls.	Mean age 65 years Excluded prior VTE.	CRP at baseline.	OR 1.6 (95% CI 1.2-2.2) for CRP in highest quintile compared to lowest quintile. Highest risk if the VTE occurred within a year of blood sampling.	Yes
Hald et al., 2011 (57)Tromso study	Cohort study; 6426 participants.	25-84 years Excluded prior VTE and if CRP >10 mg/L.	CRP at baseline, prospective follow up.	HR 1.08 per 1 SD increase in hsCRP (95% CI 0.95-1.23).No increased risk of VTE across quartiles of hsCRP (p=0.6)	No
Olson et al., 2014 (58)REGARDS	Cohort study; 30239 participants.	Caucasian and African American aged>45 years.	CRP at baseline, prospective follow up.	HR for VTE 1.25 per SD increase in log-CRP (CI 1.09-1.43)	Yes
Kunutsor et al., 2017 (59)	Cohort study;2420 participants.	Men aged 42-61 years	Baseline CRP, prospective follow up.	HR for VTE 1.17per 1 SD increase in log-CRP (CI 0.98-1.4)	Yes
Grimnes et al., 2018 (50)Tromso study	Case-crossover study;707 patients with VTE.	>25 years Excluded CRP levels taken within 2 days of VTE episode.	CRP in 90 days prior to VTE (“hazard period”) compared to CRP in four preceding 90-day “control periods”.	Median CRP58% higher in hazard period compared to control period.1 unit increase in log-transformed CRP OR 1.79 (95% CI 1.48-2.16).	Yes

VTE, venous thromboembolism; CRP, C-reactive protein; CI, confidence interval; IBD, inflammatory bowel disease; HRT, hormone replacement therapy; OR, odds ratio; HR, hazard ratio; SD, standard deviation; hsCRP, high sensitivity CRP; LETS, Leiden Thrombophilia Study; LITE, Longitudinal Investigation of Thromboembolism Etiology; CHS, Cardiovascular Healthy Study; ARIC, Atherosclerosis Risk in Communities; MAISTHRO, Main-ISar-THROmbosis; CCHS, Copenhagen City Heart Study; CGPS, Copenhagen General Population Study; HUNT-2, Nord-Trondelag Health Study 1995-1997; REGARDS, Reasons for Geographic and Racial Differences in Stroke.

Long-term population-based studies assessing baseline circulating CRP levels and future risk of VTE have shown a modest association between CRP levels and VTE (**Table 1**). A Danish study of over 10,000 participants found those with a baseline level of high sensitivity CRP (hsCRP) > 3 mg/L had a 2.3 times increased risk of VTE compared to those with levels < 1 mg/L, after adjustment for age, sex and use of statins over 16 years of follow up (44). Additionally, there was a stepwise increase in VTE risk as circulating CRP levels rose. The ARIC study of over 10,000 participants with an 8 year follow up period also found a higher baseline hsCRP level to be associated with an increased risk of VTE. The authors report a HR 1.6-2 times higher for values above versus below the 90^th^ percentile of CRP (> 8.55 mg/L), after exclusion of those with prior VTE and those with very elevated circulating CRP levels (> 20 mg/L) (54). Quist-Paulsen et al. similarly found a positive association between baseline hsCRP levels and subsequent VTE, with a 1.6-fold increased risk of VTE in those with the highest quintile versus the lowest quintile of CRP. Interestingly, they found the strongest association in individuals who experienced venous thrombosis within a year of blood sampling, suggesting direct stimulation of the coagulation system by short-term inflammatory processes (56). A meta-analysis of nine population-based studies, with 81,625 participants, found an adjusted risk estimate for VTE of 1.14 (95 % CI 1.08-1.19) per standard deviation (SD) increase in baseline CRP (59).

In contrast, a number of studies have found no effect of circulating CRP levels on VTE risk (**Table 1**). A study of over 6,000 individuals followed for 12.5 years found no increased risk of VTE per 1 SD increase in baseline hsCRP or across quintiles of CRP (57). This held true after adjusting for body-mass-index (BMI), smoking, diabetes or whether the VTE episode was provoked or unprovoked (57). They excluded those with prior VTE and those with hsCRP greater than 10 mg/L to remove any affect related to acute inflammation (57). The longitudinal Physicians Health Study (36) and Longitudinal Investigation of Thromboembolism Etiology (LITE) Study (52) also found CRP levels were not predictive of future VTE. The LITE study looked at 20,000 healthy participants without a history of VTE with a follow up time of 7.8 years (52), and found a linear association with factor VIII and vWF levels, but no association with CRP.

Considering the inconsistent and, at best, modest association of circulating CRP levels and VTE in all-comers, several studies have investigated different subgroups of patients with VTE to determine if there may be a stronger association. In this regard, obesity is a known risk factor for VTE and is associated with double the risk of VTE compared to people of normal weight (61). Several studies have now assessed the role of a range of biomarkers, including circulating CRP levels, in explaining the increased risk of VTE in this population. To date, these results have been conflicting (58, 62), but appear to suggest an association between elevated CRP levels and obesity in older individuals and females (36, 59, 60).

Patients undergoing major surgery are another group who are considered to be at high risk of developing VTE and circulating CRP levels may aid in identifying individuals at particularly high risk. A small study of 32 patients demonstrated those that developed VTE had a significantly higher increase in CRP levels on post-operative day 1 compared to their pre-operative baseline (63). The circulating CRP level was significantly correlated with platelet-monocyte aggregates, supporting the interplay between CRP and inflammation (63). CRP has also been shown to be associated with a higher risk of pre-operative DVT in individuals with closed femur fractures (64). After lower-risk surgery (such as laparoscopic cholecystectomy), CRP levels typically rise on post-operative day 7-10 but then normalize by day 30 and are not associated with VTE risk (65).

Overall, the measurement of plasma CRP does not appear to be a sensitive biomarker in predicting VTE risk in unselected patient groups. However, it may have utility in certain populations, including obese individuals, females and those undergoing major surgery. The lack of consistent association in unselected patient groups is likely related to normal intra-individual variation in circulating CRP levels, timing of CRP measurement as well as measurement differences. Lastly, the potential role of different isoforms of CRP as predictors of incident VTEs remains to be investigated.

## CRP as a predictor of VTE recurrence

A biomarker that would identify individuals at high risk of recurrence following the first unprovoked VTE would be extremely beneficial, as it would improve individualization of the duration of anticoagulation, based on predicted recurrence risk. Currently we rely on clinical prediction scores for risk of recurrent VTE, such as Vienna, DASH and HERDOO2. However, all three have deficiencies such as an inability to stratify males, and complexities meaning they are difficult to use in routine clinical practice (66–68). In this regard, there are several clinical situations where patients are recommended to continue anticoagulation indefinitely due to the high risk of recurrent VTE, such as in CAT or antiphospholipid syndrome. However, in many other situations the decision for continued anticoagulation is less clear and relies on discussion of population-based predicted recurrence risks balanced against bleeding risks and patient wishes (69). In these situations of clinical equipoise, a novel biomarker to predict recurrent VTE to allow the use of anticoagulation in only truly high-risk patients would represent a significant advance.

Several studies have shown that circulating CRP levels are higher in those with a history of VTE compared to those without VTE, and in particular that CRP is higher in those with unprovoked compared to provoked VTE (53, 55). The Leiden Thrombophilia study found patients with a prior VTE had higher CRP levels than healthy controls, when circulating CRP was measured between 6 and 18 months following acute VTE (51). However, much of this difference was driven by the fact that 11 % of VTE patients had CRP levels above the 95^th^ percentile of the control value (9.75 mg/L), and when these were excluded the effect disappeared (51). Regardless, this does suggest that ongoing systemic inflammation is more prominent among VTE patients (51). While these studies did not link the results to risk of VTE recurrence, a sub-study of the Leiden Thrombophilia Study followed those with a prior DVT and assessed circulating CRP levels at least 3 months after discontinuation of oral anticoagulation (70). They found those with an elevated D-dimer and/or CRP level had a higher risk of recurrence compared to those with low CRP and D-dimer levels with a HR of 1.9. Another prospective study assessing risk factors for recurrence after unprovoked VTE involving 229 patients similarly found elevated hsCRP levels, as well as iron deficiency, predicted VTE recurrence over the subsequent 24 months (71). These studies highlight the potential for using CRP in combination with other biomarkers or clinical prediction model to improve risk the risk stratification of this patient cohort.

## CRP as a predictor of cancer-associated VTE

CAT is a major health problem and is the leading cause of non-cancer death in cancer patients (72). Importantly, the prevention and management of VTE in this cohort is challenging due to the increased risk of both bleeding and thrombotic complications. In this regard, the identification of a novel biomarker to improve VTE risk stratification is required (72). The identification of patients at high risk of CAT is currently based on clinical risk scores such as the Khorana score (73). While the Khorana score is well-validated, a significant number (5-7 %) of VTE events still occur in those classified as low- or intermediate-risk (74). Furthermore, those with lung cancer or hematologic malignancies classified as high risk by the Khorana score have a much lower estimated risk of VTE than other cancer types and as such is less informative in these groups of patients (74). This is evidenced by the fact that using clinical risk scores alone (Khorana), VTE prophylaxis using rivaroxaban in ambulatory cancer patients did not result in a significantly lower incidence of VTE or death over 6 months of follow up, but did result in a reduction of VTE on longer follow-up (75). The studies show that there are clearly deficiencies in using clinical risk scores alone, and other ways to stratify VTE risk in patients are needed.

CRP can be produced by cells within the tumor microenvironment including stromal cells and mesenchymal-like tumor cells (76, 77). Interestingly, activated platelets, known to destabilize pCRP to the pro-inflammatory pCRP*/mCRP isoforms, have been identified as an early marker of primary tumors and metastases (78), indicating that CRP activation at the site of cancerous tissue contributes to a pro-inflammatory state in cancer patients. Accordingly, serum levels of CRP correlate with the tumor mass in patients, with high levels of CRP reflecting advanced disease and poor prognosis (76). Regarding CAT, several biomarkers have been assessed for prediction of primary VTE risk, but CRP has not been studied as extensively as other biomarkers such as D-dimer, leukocyte and platelet count. A small study of 35 patients found that the combination of hsCRP, D-dimer and the clinical Wells score could predict the incidence of acute DVT in cancer patients (79). A study of 507 cancer patients in Germany, of which 60 developed a new VTE, found that those with VTE had higher circulating CRP levels in the 30 days prior to the VTE episode than those without any VTE in the follow up period (80). In particular, an elevated CRP level > 5 mg/L was linked with the occurrence of VTE (80). The Vienna Cancer and Thrombosis Study (CATS) followed 705 cancer patients for two years after an initial blood sample (81). They found the cumulative probability of developing VTE after 12 months was 11.7 % in those with a higher circulating CRP level (above the 75^th^ percentile), compared to 4.9 % for those with lower CRP levels (81). After adjusting for age, gender, chemotherapy, surgery, radiotherapy and BMI, the HR of VTE per doubling of CRP was 1.1. In support, the authors report that circulating CRP level was clearly associated with a worse survival probability (81).

Biomarkers such as CRP may also further enable identification of those at lower risk of recurrence after a first CAT – potentially allowing the cessation of anticoagulation and thus mitigating the accompanying bleeding risk. Current recommendations for CAT are to instigate anticoagulation which continues indefinitely while cancer is active, or the individual is receiving anti-cancer therapy unless there are contraindications (82). To determine if circulating CRP levels, in addition to D-dimer, could predict VTE risk after cessation of anticoagulation after CAT, Jara-Palomares et al. assessed these parameters 21 days after anticoagulation cessation (83). They found a hsCRP level > 4.5 mg/L could predict VTE recurrence and would allow for 66 % of patients to stop anticoagulation based on the hsCRP level at this time point (83). The 6-month VTE recurrence rate was 8.8 %, which decreased to 1.5 % if the 21-day hsCRP level was < 4.5 mg/L (83). CRP was also evaluated as part of the CATCH trial, which compared tinzaparin with warfarin in the treatment of 900 patients with acute CAT – they found a mean circulating CRP level of 50.3 mg/L at the time of acute VTE, and that the uppermost quartile of CRP (> 75 mg/L) was associated with recurrent VTE in univariate analysis (RR 2.3) which was maintained after adjustment for competing risks such as history of VTE or metastatic disease (84).

Overall, this evidence suggests that individuals with cancer and a higher circulating CRP level have an increased risk of developing a first thrombosis, and a higher risk of recurrence following treatment for CAT if the CRP level remains high after cessation of anticoagulation.

## CRP as a predictor of VTE in COVID-19

From an early stage in the coronavirus disease 2019 (COVID-19) pandemic, VTE was seen as a frequent ‘immunothrombotic’ complication in affected individuals (85, 86). Indeed, thrombotic complications have been reported to affect up to half of critically ill COVID-19 patients admitted to the intensive care unit and 7.9 % of hospitalized COVID-19 patients, significantly higher than rates seen in other respiratory illnesses (87, 88).

Elevation in circulating CRP levels in the context of COVID-19 has been shown to be associated with VTE. A case-control study of 256 non-critically ill and 144 critically ill COVID-19 patients in the USA found a circulating CRP level of > 100 mg/L at presentation to be associated with thrombotic complications with an adjusted OR of 3.04 (89). A number of subsequent retrospective studies have found higher CRP levels in those with VTE compared to those without VTE, with the positive predictive value of CRP enhanced when combined with D-dimer (90, 91). A prospective study confirmed circulating CRP levels on admission to be an independent predictor of incident pulmonary embolism during hospitalization with COVID-19 (92). Therefore, it appears CRP is useful in predicting VTE in the context of COVID-19, with D-dimer further improving risk assessment, however larger prospective studies are required to confirm the association.

Considering elevations in CRP levels have been associated with acute thrombotic episodes in the general population, it follows that it would also be associated with VTE in COVID-19. Furthermore, our current understanding of the pathophysiology of thrombosis in COVID-19 highlights important roles of platelet activation, endothelial inflammation and NETosis in the setting of COVID-19 infection (85). Recent clinical trials have suggested anti-thrombotic therapies may improve outcomes in patients with COVID-19, but at the expense of more bleeding (93). Therefore, novel approaches to target the deleterious thromboinflammatory response associated with COVID-19 infection remains an urgent unmet clinical need with the ongoing global COVID-19 pandemic.

## Summary and perspectives

The data for circulating CRP levels as a predictor for VTE in the unselected general population remains mixed. However, in patients with a prior history of VTE, obesity, cancer and COVID-19 the cumulative data supports that elevations of CRP may aid in the prediction of VTE risk. Although these studies do not imply causality, further investigations are required to unravel whether circulating CRP plays a direct role in the pathogenesis of VTE. The potential pathogenic role of CRP in cardiovascular disease has spurred much debate over nearly two decades; however, it is clear that CRP binding to damaged or inflamed tissue can amplify tissue damage and inflammation, and is now implicated in the pathogenesis of a number of thromboinflammatory diseases such as ischemia/reperfusion injury. In contrast, the infusion of purified pCRP into healthy adults elicits no such inflammatory response (7). Therefore, it is tempting to speculate that CRP’s role in VTE is similar. That is, in the context of concurrent inflammation, as seen in obesity, cancer and COVID-19, a fertile environment is present for the ‘activation’ of CRP, which can then exacerbate the thromboinflammatory processes involved in VTE formation. In this regard, future studies are required to understand whether circulating CRP plays a direct role mediating VTE. Moreover, the assessment of CRP isoforms in human venous thrombosis will provide further insights regarding the potential pathogenic role of CRP in VTE. A range of therapeutic strategies directed at inhibiting the pro-inflammatory effects of CRP isoforms have been developed that display profound anti-inflammatory effects in cardiac and renal ischemia/reperfusion injury, and transplant rejection. Therefore, addressing the role of CRP, particularly its dissociation in pro-inflammatory/pro-aggregatory isoforms, in VTE is fundamental given the ongoing interest in developing novel therapeutic/preventative strategies to target thromboinflammation. Such strategies may hold promise for a range of diseases including VTE prevention/therapy and the treatment of COVID-19.

## Author contributions

CD, JZ, HS, KP, JM conceived and wrote the manuscript. SE, TN, CM, MP co wrote and edited the manuscript. All authors contributed to the article and approved the submitted version.

## Funding

HS is supported by a Monash University RTP Scholarship and Wheaton Family Scholarship. KP is supported by a National Health and Medical Research Council (NHMRC) Senior Principal Research Fellowship. JM is supported by a NHMRC and Heart Foundation Early Career Fellowship. Funding from the Victorian Government Operational Infrastructure Support Scheme to St Vincent’s Institute is acknowledged. MP is a National Health and Medical Research Council of Australia Leadership Fellow.

## Conflict of interest

The authors declare that the research was conducted in the absence of any commercial or financial relationships that could be construed as a potential conflict of interest.

## Publisher’s note

All claims expressed in this article are solely those of the authors and do not necessarily represent those of their affiliated organizations, or those of the publisher, the editors and the reviewers. Any product that may be evaluated in this article, or claim that may be made by its manufacturer, is not guaranteed or endorsed by the publisher.

## Abbreviations

BMI, Body-mass-index; CAMs, Cell adhesion molecules; CAT, Cancer-associated thrombosis; CI, Confidence interval; COVID-19, Coronavirus disease 19; CRP, C-reactive protein; hsCRP, High-sensitivity CRP; mCRP, Monomeric CRP; pCRP, Pentameric CRP; pCRP*, Neoepitope expressing pCRP; DVT, Deep vein thrombosis; EC, Endothelial cells; HR, Hazard ratio; HRT, Hormone replacement therapy; IBD, Inflammatory bowel disease; NETs, Neutrophil extracellular traps; MV, Microvesicles; OR, Odds ratio; PC, Phosphocholine; RBC, Red blood cells; RR, Relative risk; TF, Tissue factor; VTE, Venous thromboembolism; vWF, von Willebrand Factor.
